# Crystalline retinopathy in a child with hyperoxaluria type 1: Ultrawide field imaging before and after treatment^☆^

**DOI:** 10.1016/j.ajoc.2026.102531

**Published:** 2026-01-30

**Authors:** Ysé Borella, Romane Boinet, Dominique Bremond-Gignac, Alejandra Daruich

**Affiliations:** a Ophthalmology Department, Necker-Enfants Malades University Hospital, AP-HP, OPHTARA Rare Eye Disease Center, Paris Cité University, 149 Rue de Sèvres, 75015, Paris, France; b INSERM, UMRS1138, Team 17, From Physiopathology of Ocular Diseases to Clinical Development, Sorbonne Paris Cité University, Centre de Recherche des Cordeliers, Paris, France

**Keywords:** Crystalline retinopathy, Hyperoxaluria type 1, RNA interference therapy

## 1 Case report

**Fig. 1:**
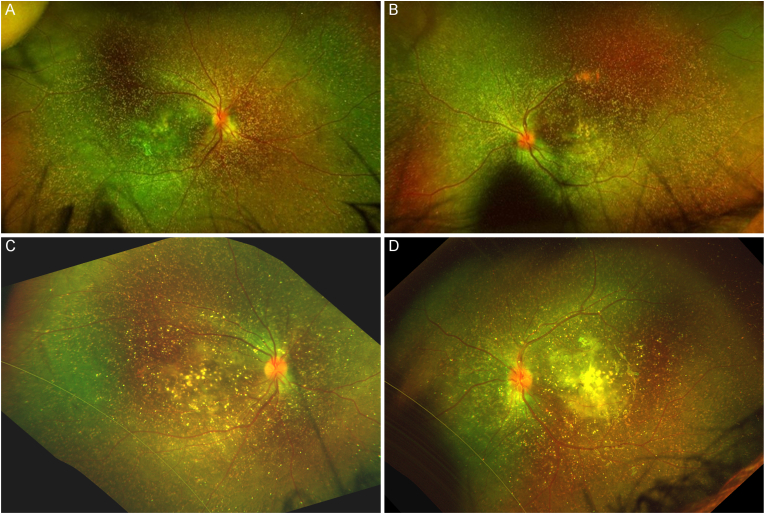
Ultrawide field imaging of crystalline retinopathy in a one-year-old boy with hyperoxaluria type 1, before and one year after treatment initiation with RNA interference retinopathy. A–B: One-year-old boy before therapy (A: right eye, B: left eye). C–D: At the age of two years old, after one year of treatment (C: right eye, D: left eye). After one year of treatment, we observed that peripheral crystals were more faint compared to before treatment initiation, however a macular scar developed in both eyes, more pronounced in the left eye.

A one-year-old boy born from 2nd degree blood-related parents was referred to our department for fundus examination for suspicion of hyperoxaluria type 1. He was diagnosed with terminal renal insufficiency of unknown origin at the age of six months following status epilepticus, and underwent peritoneal dialysis from this age. Fundus examination showed diffuse crystalline retinopathy (yellow flecks), except in the far temporal periphery, in both eyes (Fig. 1A–B). Slit lamp examination, intraocular pressure, electroretinogram, refraction and visual behavior were normal. Genetic testing confirmed primitive type 1 hyperoxaluria with homozygous mutation of AGXT (c.919del; p. Leu307fs). The child was treated with subcutaneous ribonucleic acid (RNA) interference therapy (lumasiran, 6 mg/kg every month for three months, then every three months)^1^ and underwent renal transplant in his second year of life. After one year of treatment, blood oxalate levels were controlled between 15 and 20 μmol/L one year after the initiation of the treatment, and we observed in this young boy that peripheral crystals were more faint (Fig. 1C–D) compared to the fundus examination performed before treatment initiation, however a macular scar was already present in left eye (Fig. 1, D). Visual behavior of the child did not show any significant change, albeit difficult to assess at this young age.

## 2 Discussion

Hyperoxaluria is caused by altered glyoxylate metabolism leading to oxalate overproduction.^2^ Ocular manifestations are highly polymorphic and not related to the genotype. Severity of the ocular manifestation is linked to macular scaring following oxalate deposits, and to the earliness of the onset.^3^ Renal or liver failure can negatively impact systemic prognosis.^2^ However, in front of this good response to medical treatment with no adverse effects in our patient, it was decided not to perform liver transplantation, that can be associated with significant risks. The irreversibility of crystalline retinopathy is debated in the literature. One team reported the regression of retinal crystals after renal transplantation in 6-month-old patient due to blood hyperoxalosis. However, this patient showed a normal glyoxylate metabolism activity on the hepatic biopsy, suggesting another mechanism of hyperoxalosis other than hyperoxaluria type 1 or type 2 and a potential different metabolism of the retinal crystal than in type 1 hyperoxaluria patients.^4^ In our patient, the peripheral crystals became more faint after one year of treatment. However, macular scarring also developed. Macular scarring can be present in up to 2/3 of the patients with early infantile disease.^3^ As the natural history of retinal oxalate crystals is still unclear in the literature, it is unclear whether the macular scarring was enhanced by the treatment itself or if it was solely the result of the disease's progression.

## 3 Conclusion

RNA interference therapy for treating hyperoxaluria type 1 may help to improve the crystalline retinopathy, but could also promote the evolution to macular scarring.

## CRediT authorship contribution statement

**Ysé Borella:** Writing – original draft, Validation, Methodology, Investigation, Formal analysis, Data curation, Conceptualization. **Romane Boinet:** Writing – review & editing, Investigation, Data curation. **Dominique Bremond-Gignac:** Writing – review & editing, Validation, Supervision. **Alejandra Daruich:** Writing – review & editing, Validation, Supervision, Project administration, Methodology, Investigation, Data curation, Conceptualization.

## Patient consent

Written consent to publish this case has not been obtained. This report does not contain any personal identifying information. Informed consent was obtained by a non-opposition institutional CRMR OPHTARA (Centre de Reference Maladies Rares en Ophtalmologie, accredited by French Health Ministry and Europe ERN-Eye) consent.

## Authorship

All authors attest that they meet the current ICMJE criteria for Authorship.

## Funding

No funding or grant support

## Declaration of competing interest

The following authors have no financial disclosures: YB, RB, DBG, AD.
